# Theorems on Positive Data: On the Uniqueness of NMF

**DOI:** 10.1155/2008/764206

**Published:** 2008-03-25

**Authors:** Hans Laurberg, Mads Græsbøll Christensen, Mark D. Plumbley, Lars Kai Hansen, Søren Holdt Jensen

**Affiliations:** ^1^Department of Electronic Systems, Aalborg University, Niels Jernes Vej 12, 9220 Aalborg, Denmark; ^2^Department of Electronic Engineering, Queen Mary, University of London, Mile End Road, London E1 4NS, UK; ^3^Department of Informatics and Mathematical Modeling, Technical University of Denmark, Richard Petersens Plads, Building 321, 2800 Lyngby, Denmark

## Abstract

We investigate the conditions for which nonnegative matrix factorization (NMF) is unique and introduce several theorems which can determine whether the decomposition is in fact unique or not. The theorems are illustrated by several examples showing the use of the theorems and their limitations. We have shown that corruption of a unique NMF matrix by additive noise leads to a noisy estimation of the noise-free unique solution. Finally, we use a stochastic view of NMF to analyze which characterization of the underlying model will result in an NMF with small estimation errors.

## 1. Introduction

Large quantities of positive data occur in research areas such as music analysis, text analysis, image analysis, and probability theory. Before deductive science is applied to large quantities of data, it is often appropriate to reduce data by preprocessing, for example, by matrix rank reduction or by feature extraction. Principal component analysis is an example of such preprocessing. When the original data is nonnegative, it is often desirable to preserve this property in the preprocessing. For example, elements in a power spectrogram, probabilities, and pixel intensities should still be nonnegative after the processing to be meaningful. This has led to the construction of algorithms for rank reduction of matrices and feature extraction generating nonnegative output. Many of the algorithms are related to the nonnegative matrix factorization (NMF) algorithm proposed by Lee and Seung [1, 2]. NMF algorithms factorize a nonnegative matrix **V** ∈ ℝ_+_^*n*×*m*^ or **R** ∈ ℝ_+_^*n*×*m*^ into two nonnegative matrices **W** ∈ ℝ_+_^*n*×*r*^ and **H** ∈ ℝ_+_^*r*×*m*^:

(1) V≈R=WH.

There are no closed-form solutions to the problem of finding **W** and **H** given a **V**, but Lee and Seung [1, 2] proposed two computationally efficient algorithms for minimizing the difference between **V** and **WH** for two different error functions. Later, numerous other algorithms have been proposed (see [3]).

An interesting question is whether the NMF of a particular matrix is unique. The importance of this question depends on the particular application of NMF. There can be two different viewpoints when using a model like NMF—either one can believe that the model describes nature and that the variables **W** and **H** have a physical meaning or one can believe that the model can capture the part of interest even though there is not a one-to-one mapping between the parameters and the model, and the physical system. When using NMF, one can wonder whether **V** is a disturbed version of some underlying **WH** or whether the data is constructed by another model or, in other words, a ground truth **W** and **H** does exist. These questions are important in evaluating whether or not it is a problem that there is another NMF solution, **W**′**H**′, to the same data, that is,

(2) $$V\approx R=WH=W^{\prime }H^{\prime }.$$

If NMF is used even though the data is not assumed to be generated by (1), it may not be a problem that there are several other solutions. On the other hand, if one assumes that a ground truth exists, it may be a problem if the model is not detectable, that is, if it is not possible to find **W** and **H** from the data matrix **V**.

The first articles on the subject was two correspondences between Berman and Thomas. In [4] Berman asked for what amounts to a simple characterization of the class of nonnegative matrices **R** for which an NMF exists. As we shall see, the answer by Thomas [5] can be transferred into an NMF uniqueness theorem.

The first article investigating the uniqueness of NMF is Donoho and Stodden [6]. They use convex duality to conclude that in some situations, where the column vectors of **W** “describe parts,” and for that reason are nonoverlapping and thereby orthogonal, the NMF solution is unique.

Simultaneously with the development of NMF, Plumbley [7] worked with nonnegative independent component analysis where one of the problems is to estimate a rotation matrix **Q** from observations on the form **Qs**, where **s** is a nonnegative vector. In this setup, Plumbley investigates a property for a nonnegative independent and identically distributed (i.i.d.) vector **s** such that **Q** can be estimated. He shows that if the elements in **s** are grounded and a sufficiently large set of observations is used, then **Q** can be estimated. The uniqueness constraint in [7] is a statistical condition of **s**.

The result in [7] is highly relevant to the NMF uniqueness due to the fact that in most cases new NMF solutions will have the forms **WQ** and **Q** ^−1^ **H** as described in Section 3. By using Plumbley's result twice, a restricted uniqueness theorem for NMF can be constructed.

In this paper, we investigate the circumstances under which NMF of an observed nonnegative matrix is unique. We present novel necessary and sufficient conditions for the uniqueness. Several examples illustrating these conditions and their interpretations are given. Additionally, we show that NMF is robust to additive noise. More specifically, we show that it is possible to obtain accurate estimates of **W** and **H** from noisy data when the generating NMF is unique. Lastly, we consider the generating NMF as a stochastic process and show that particular classes of such processes almost surely result in unique NMFs.

This paper is structured as follows. Section 2 introduces the notation, some definitions, and basic results. A precise definition and two characterizations of a unique NMF are given in Section 3. The minimum constraints of **W** and **H** for a unique NMF are investigated in Section 4. Conditions and examples of a unique NMF are given in Section 5. In Section 6, it is shown that in situations where noise is added to a data matrix with a unique NMF, it is possible to bound the error of the estimates of **W** and **H**. A probabilistic view on the uniqueness is considered in Section 7. The implication of the theorems is discussed in Section 8, and Section 9 concludes the paper.

## 2. Fundamentals

We will here introduce convex duality that will be the framework of the paper, but first we shall define the notation to be used. Nonnegative real numbers are denoted as ℝ_+_, ||⋅||_*F*_ denotes the Frobenius norm, and span(⋅) is the space spanned by the set of vectors. Each type of variables has its own font. For instance, a scalar is denoted *x*, a column vector is denoted **x**, a row vector is denoted by $\underline{x}$, a matrix is denoted by **X**, a set is denoted by *𝒳*, and a random variable is denoted by ***𝒳***. Moreover, **x** *_i_^j^* is the *i*th index of the vector **x** ^*j*^. When a condition for a set is used to describe a matrix, it is referring to the set of column vectors in the matrix. The NMF is symmetric in **W** ^*T*^ and **H**, so the theorems for one matrix may also be used for the other.

In the paper, we make a geometric interpretation of the NMF similar to that used in both [5, 6]. For that, we need the following definitions.

**Definition 1.**

The *positive span* is given by span^+^(**b** ^1^,…, **b** ^*d*^) = {**v** = ∑_*i*_ **b** ^*i*^ **a**_*i*_ ∣ **a** ∈ ℝ_+_^*d*^}.

In some literature, the positive span is called the conical hull.

**Definition 2.**

A set ***𝒜*** is called a *simplicial cone* if there is a set ***ℬ*** such that ***𝒜*** = span^+^ (***ℬ***). The *order* of a simplicial cone ***𝒜*** is the minimum number of elements in ***ℬ***.

**Definition 3.**

The *dual* to a set ***𝒜***, denoted 𝒜*, is given by 𝒜* = {**v** ∣ **v** ^*T*^ **a** ≥ 0 for all **a** ∈ ***𝒜***}.

The following lemma is easy to prove and will be used subsequently. For a more general introduction to convex duality, see [8].

**Lemma 1.**

(a) *If **𝒳*** = span^+^(**b** ^1^ …,**b** ^*d*^), *then* **y** ∈ 𝒳* * if and only if* **y** ^*T*^ **b** ^*n*^ ≥ 0 *for all* *n*.

(b) *If **𝒳*** = span^+^(**B** ^*T*^) *and* **B** ^*T*^ = [**b** ^1^,…, **b** ^*d*^] *is invertible, then* 𝒳* = span^+^(**B** ^−1^).

(c) *If **𝒴*** ⊆ ***𝒳***, then 𝒳* ⊆ 𝒴*.

(d) *If **𝒴*** and ***𝒳*** *are closed simplicial cones and **𝒴*** ⊂ ***𝒳***, then 𝒳* ⊂ 𝒴*.

## 3. Dual Space and the NMF

In this section, our definition of unique NMF and some general conditions for unique NMF are given. As a starting point, let us assume that both **W** and **H** have full rank, that is, *r* = rank(**R**).

Let **W**′ and **H**′ be any matrices that fulfil, **R** = **WH** = **W**′**H**′. Then, span(**W**) = span(**R**) = span(**W**′). The column vectors of **W** and **W**′ are therefore both bases for the same space and as a result there exists a basis shift matrix **Q** ∈ ℝ^*r*×*r*^ such that **W**′ = **WQ**. It follows that **H**′ = **Q** ^−1^ **H**. Therefore, all NMF solutions where *r* = rank(**R**), are of the form **R** = **WQQ** ^−1^ **H**. In these situations, the ambiguity of the NMF is the **Q** matrix. Note that if *r* > rank(**R**) the above arguments are not valid because rank(**W**) can differ from rank(**W**′) and thereby span(**W**) ≠ span(**W**′).

**Example 1.**

The following is an example of an ℝ_+_^4×4^ matrix of rank 3, where there are two NMF solutions but no **Q** matrix to connect the solutions

(3) $$\left[\begin{array}{cccc} 1 & 1 & 0 & 0\\ 1 & 0 & 1 & 0\\ 0 & 1 & 0 & 1\\ 0 & 0 & 1 & 1 \end{array}\right]=R=\underset{W}{\underset{︸}{R}}\underset{H}{\underset{︸}{I}}=\underset{W^{\prime }}{\underset{︸}{I}}\underset{H^{\prime }}{\underset{︸}{R}}.$$

We mention in passing that Thomas [5] uses this matrix to illustrate a related problem. This completes the example.

**Lemma 2 (Minc [9, Lemma 1.1]).**

*The inverse* *of a nonnegative matrix is nonnegative if and only if it is a scaled permutation*.

Lemma 2 shows that all NMF solutions on the forms **WQ** and **Q** ^−1^ **H**, where **Q** is a scaled permutation, are valid, and thereby that NMF only can be unique up to a permutation and scaling. This leads to the following definition of unique NMF in this paper.

**Definition 4.**

A matrix **R** has a *unique NMF* if the ambiguity is a permutation and a scaling of the columns in **W** and rows in **H**.

The scaling and permutation ambiguity in the uniqueness definition is a well-known ambiguity that occurs in many blind source separation problems. With this definition of unique NMF, it is possible to make the following two characterizations of the unique NMF.

**Theorem 1.**

*If r* = *rank*(**R**), *an NMF is unique if and only if the positive orthant is the only r*-*order simplicial cone **𝒬** such that* span^+^(**W** ^*T*^) ⊆ ***𝒬*** ⊆ span^+^(**H**)*.

**Proof.**

The proof follows the analysis of the **Q** matrix above in combination with Lemma 1(b). The theorem can also be proved by following the steps of the proof in [5].

**Theorem 2 (see [6]).**

*The NMF is unique if and only if there is only one r*-*order simplicial cone **𝒬** such that* span^+^(**R**) ⊆ ***𝒬*** ⊆ ***𝒫***, *where **𝒫** is the positive orthant*.

**Proof.**

The proof follows directly from the definitions.

The first characterization is inspirited by [5] and the second characterization is implicit introduced in [6]. Note that the two characterizations of the unique NMF analyze the problem from two different viewpoints. Theorem 1 takes a known **W** and **H** pair as starting point and looks at the solution from the “inside,” that is, the *r*-dimensional space of row vectors in **W** and column vectors in **H**. Theorem 2 looks at the problem from the “outside,” that is, the *n*-dimensional column space of **R**.

## 4. Matrix Conditions

If **R** = **WH** is unique, then both **W** and **H** have to be unique, respectively, that is, there is only one NMF of **W** and one of **H**, namely, **W** = **WI** and **H** = **IH**. In this section, a necessary condition for **W** and **H** is given and a sufficient condition is shown.

The following definition will be shown to be a necessary condition for both the set of row vectors in **W** and column vectors in **H**.

**Definition 5.**

A set *𝒮* of vectors in ℝ_+_^*d*^ is called *boundary close* if for all *j* ≠ *i* and *k* > 0 there is an element **s** ∈ *𝒮* such that

(4) $$s_{j}<ks_{i}.$$

In the case of closed sets, the boundary close condition is that **s**_*j*_ = 0 and **s**_*i*_ ≠ 0. In this section, the sets will be finite (and therefore closed), but in Section 7 the general definition above is needed.

**Theorem 3.**

*The set of row vectors in* **W** *has to be boundary close for the corresponding NMF to be unique*.

**Proof.**

If the set of row vectors in **W** are not boundary close, there exist indexes *j* ≠ *i* and *k* > 0 such that the *j*th element is always more than *k* times larger than the *i*th element in the row vectors in **W**. Let ***𝒬*** = span^+^(**q** ^1^,…, **q** ^*r*^), where

(5) $$q^{n}=\{\begin{array}{ll} e^{i}+ke^{j} & \text{if}\,\;\;n=i,\\ e^{n} & \text{otherwise}, \end{array}$$

and **e** ^*n*^ denotes the *n*th standard basis vector. This set fulfils the condition span^+^(*W^T^*) ⊆ ***𝒬*** ⊂ ***𝒫*** and we therefore, using Theorem 1, conclude that the NMF cannot be unique.

That not only the row vectors of **W** with small elements determine the uniqueness can be seen from the following example.

**Example 2.**

The following is an example where $\overset{¯}{W}$ is not unique but $W=\left[\begin{array}{ccc} & \overset{¯}{W} & \\ 3 & 1 & 1 \end{array}\right]$ is.

Let

(6) $$\overset{¯}{W}=\left[\begin{array}{ccc} 0 & 1 & 1\\ 1 & 0 & 1\\ 1 & 1 & 0 \end{array}\right].$$

**Figure 1:**
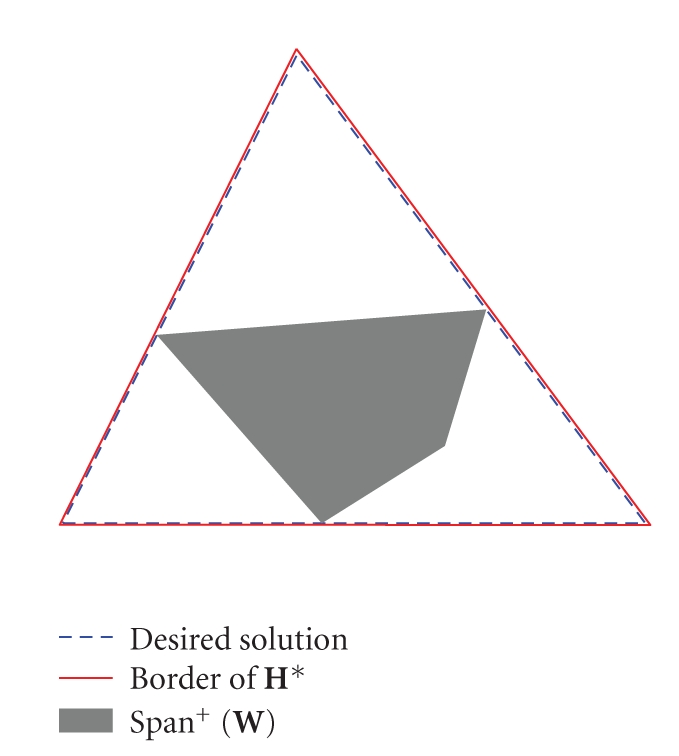
A three-dimensional space is scaled such that the vectors are in the hyper plane: {**p** ∣ [1 1 1]**p** = 1}. By the mapping to the hyper plane, a plane in ℝ^3^ is mapped to a line and a simplicial cone is mapped to an area. In the figure, it can be observed that the dashed triangle (desired solution) is the only triangle (third-order simplicial cone) that contains the shaded area (positive span of **W**) while being within the solid border (the dual of **H**). The NMF can be concluded to be unique by Theorem 1.

Here $\overset{¯}{W}$ is boundary close but not unique since $\overset{¯}{W}=\overset{¯}{W}I=I\overset{¯}{W}$. The uniqueness of $W=\left[\begin{array}{ccc} & \overset{¯}{W} & \\ 3 & 1 & 1 \end{array}\right]$ can be verified by plotting the matrix as shown in Figure 1, and observe that the conditions of Theorem 1 are fulfilled. This completes the example.

In three dimensions, as in Example 2, it is easy to investigate whether a boundary close **W** is unique—if **W** = **W**′**H**′, then **H**′ can only have two types of structure: either the trivial (desired) solution where **H**′ = **I** or a solution where only the diagonal of **H**′ is zero. In higher dimensions, the number of combinations of nontrivial solutions increases and it becomes more complicated to investigate all possible nontrivial structures. For example, if $\overset{¯}{W}$ is the matrix from Example 2, then the matrix

(7) $$\overset{˜}{W}=\left[\begin{array}{cc} \overset{¯}{W} & 0\\ 0 & \overset{¯}{W} \end{array}\right]$$

is boundary close and can be decomposed in several ways, for example,

(8) $$\begin{array}{cc} \overset{˜}{W} & =\left[\begin{array}{cc} I & 0\\ 0 & \overset{¯}{W} \end{array}\right]\left[\begin{array}{cc} \overset{¯}{W} & 0\\ 0 & I \end{array}\right]\\ & =\left[\begin{array}{cc} \overset{¯}{W} & 0\\ 0 & I \end{array}\right]\left[\begin{array}{cc} I & 0\\ 0 & \overset{¯}{W} \end{array}\right]\\ & =\left[\begin{array}{cc} I & 0\\ 0 & I \end{array}\right]\left[\begin{array}{cc} \overset{¯}{W} & 0\\ 0 & \overset{¯}{W} \end{array}\right]. \end{array}$$

Instead of seeking necessary and sufficient conditions for a unique **W**, a sufficient condition not much stronger than the necessary is given. In this sufficient condition, we only focus on the row vectors of **W** with a zero (or very small) element.

**Definition 6.**

A set of vectors ***𝒮*** in ℝ_+_^*d*^ is called *strongly boundary close* if it is boundary close, and there exists a *z* > 0 and a numbering of the elements in the vectors such that for all *k* > 0 and *n* ∈ {1,…, *d* − 1} there are *d* − *n* vectors from ***𝒮***, {**s** ^1^,…, **s** ^*d*−*n*^} that fulfil the following:

1. **s** *_n_^j^* < *k* ∑_*i*>*n*_ **s** *_i_^j^* for all *j*; and
2. *κ*_2_([**b** ^1^,…, **b** ^*d*−*n*^]) ≤ *z*, where *κ*_2_(⋅) is the “condition number” of the matrix defined as the ratio between the largest and smallest singular values [10, page 81], **b** *^j^* = **P** *_n_* **s** *^j^* and **P**_*n*_ ∈ ℝ^*d*−*n*×*d*^ is a projection matrix that picks the *d* − *n* last element of a vector in ℝ^*d*^.

**Theorem 4.**

*If* span^+^(**W** ^*T*^) * is strongly boundary close, then* **W** *is unique*.

The proof is quite technical and is therefore given in the Appendix. The most important thing to notice is that the necessary condition in Theorem 3 and the sufficient conditions in Theorem 4 are very similar. The first item in the strongly boundary close definition states that there have to be several vectors with small value. The second item ensures that the vectors with small value are linear independent in the last elements.

## 5. Uniqueness of **R**

In this section, a condition for unique **V** is analyzed. First, Example 3 is used to investigate when a strongly boundary close **W** and **H** pair is unique. The section ends with a constraint for **W** and **H** that results in a unique NMF.

**Example 3.**

This is an investigation of uniqueness of **R** when **W** and **H** are given as

(9) $$\begin{array}{ll} H & =[\begin{array}{cccccc} \alpha & 1 & 1 & \alpha & 0 & 0\\ 1 & \alpha & 0 & 0 & \alpha & 1\\ 0 & 0 & \alpha & 1 & 1 & \alpha \end{array}],\\ W & =H^{T}, \end{array}$$

where 0 < *α* < 1. Both **W** and **H** are strongly boundary close and the *z* parameter can be calculated as

(10) $$\begin{array}{ll} z & =\kappa_{2}([b^{1},\ldots ,b^{d-n}])\\ & =\kappa_{2}\left([\begin{array}{cc} \alpha & 1\\ 1 & \alpha \end{array}]\right)=\frac{1+\alpha }{1-\alpha }. \end{array}$$

**Figure 2:**
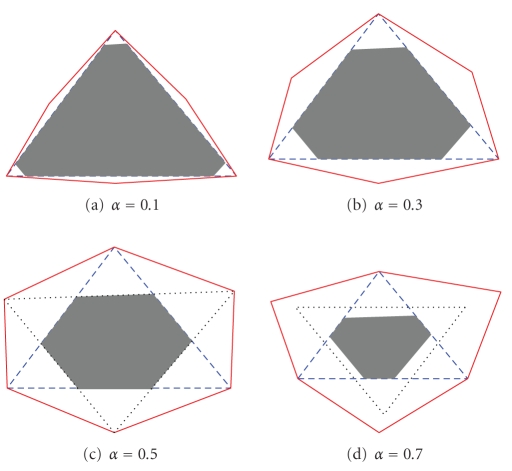
The figure shows data constructed as in Example 3 and plotted in the same manner as in Figure 1, that is, the dashed triangle is the desired solution, the solid line is the border of the dual of **H**, and the shaded area is the positive span of **W**. It can be seen that the NMF is unique when *α* equals 0.1 or 0.3 but not when *α* equals 0.5 or 0.7. In the cases where the NMF is not unique, an alternative solution is shown with a dotted line.

The equation above shows that small *α* will result in a *z* close to one and an *α* close to one results in a large *z*. In Figure 2, the matrix **R** = **WH** is plotted for *α* ∈ {0.1, 0.3, 0.5, 0.7}. The dashed line is the desired solution and is repeated in all figures. It is seen that the shaded area span^+^(**W** ^*T*^) is decreasing when *α* increases, and that the solid border span^+^(**H**)* increases when *α* increases. For all *α*-values, both the shaded area and the solid border intersect with the dashed triangle. Therefore, it is not possible to get another solution by simply increasing/decreasing the desired solution. The figure shows that the NMF is unique for *α* ∈ {0.1, 0.3} and not unique for *α* ∈ {0.5, 0.7} where the alternative solution is shown by a dotted line. That the NMF is not unique for *α* ∈ {0.5, 0.7} can also be verified by selecting the **Q** to be the symmetric orthonormal matrix

(11) $$Q=Q^{T}=Q^{-1}=\frac{1}{3}\left[\begin{array}{ccc} -1 & 2 & 2\\ 2 & -1 & 2\\ 2 & 2 & -1 \end{array}\right],$$

and see that both **WQ** and **Q** ^−1^ **H** are nonnegative. If *α* = 0.3, then the matrix **R** is given by

(12) $$R=\frac{1}{100}\left[\begin{array}{cccccc} 109 & 60 & 30 & 9 & 30 & 100\\ 60 & 109 & 100 & 30 & 9 & 30\\ 30 & 100 & 109 & 60 & 30 & 9\\ 9 & 30 & 60 & 109 & 100 & 30\\ 30 & 9 & 30 & 100 & 109 & 60\\ 100 & 30 & 9 & 30 & 60 & 109 \end{array}\right].$$

This shows that **R** needs no zeros for the NMF to be unique. This completes the example.

In the example above, **W** equals **H** ^*T*^ and thereby fulfils the same constraints. In many applications, the meaning of **W** and **H** differs, for example, in music analysis where the column vectors of **W** are spectra of notes and **H** is a note activity matrix [11].

Next, it is investigated how to make an asymmetric uniqueness constraint.

**Definition 7.**

A set of vectors in ℝ^*d*^ is called *sufficiently spread* if for all *j* and *k* > 0, there is an element **s** ∈ *𝒮* such that

(13) $$s_{j}>k\underset{i\neq j}{\sum }s_{i}.$$

Note that in the definition for sufficiently spread set the *j*th element is larger than the sum in contrast to the strongly boundary close definition where the *j*th element is smaller than the sum.

**Lemma 3.**

*The dual space* *of a sufficiently spread set is the positive orthant*.

**Proof.**

A sufficiently spread set is nonnegative and the positive orthant is therefore part of the dual set for any sufficiently spread set. Let **b** be a vector with a negative element in the *j*th element and select

(14) $$k=\frac{\sum_{i\neq j}\;|\;b_{i}\;|\;}{-b_{j}}.$$

In any sufficiently spread set, an **s** exists, such that **s**_*j*_ > *k*∑_*i*≠*j*_s_*i*_ and therefore

(15) $$\begin{array}{ll} s^{T}b & =s_{j}b_{j}+\underset{i\neq j}{\sum }s_{i}b_{i}\leq s_{j}b_{j}+(\underset{i\neq j}{\sum }s_{i})(\underset{i\neq j}{\sum }|b_{i}|)\\ & =-b_{j}(-s_{j}+k\underset{i\neq j}{\sum }s_{i})<0. \end{array}$$

The **b** is therefore not in the dual to any sufficiently spread set.

In the case of finite sets, the sufficiently spread condition is the same as the requirement for a scaled version of all the standard basis vectors to be part of the sufficiently spread set. It is easy to verify that a sufficiently spread set also is strongly boundary close and that the *z* parameter is one.

**Theorem 5.**

*If a pair* [**W** ^*T*^, **H**] *is sufficiently spread and strongly boundary close, then the NMF of* **R** = **WH** *is unique*.

**Proof.**

Lemma 3 states that the dual set of a sufficiently spread set is the positive orthant,

(16) $${\text{span}}^{+}{\left(H\right)}^{*}=𝒫={\text{span}}^{+}\,{\left(I\right)}^{*}.$$

Theorem 4 states that **WI** is unique and by using (16) and Theorem 1 we conclude that **R** = **WH** is unique.

Theorem 5 is a stronger version of the results of Donoho and Stodden [6, Theorem 1]. Theorem 1 in [6] also assumes that **H** is sufficiently spread, but the condition for **W** ^*T*^ is stronger than the strongly boundary close assumption.

## 6. Perturbation Analysis

In the previous sections, we have analyzed situations with a unique solution. In this section, it is shown that in some situations the nonuniqueness can be seen as estimation noise on **W** and **H**. The error function that describes how close an estimated [**W**′,**H**]′ pair is to the true [**W**,**H**] pair is

(17) $$J_{\left(W,H\right)}(W^{\prime },H^{\prime })=\underset{P,D}{\min}(∥{W-W^{\prime }(DP)∥}_{F}+{∥H-{\left(DP\right)}^{-1}H^{\prime }∥}_{F}),$$

where **P** is a permutation matrix and **D** is a diagonal matrix.

**Theorem 6.**

*Let* **R** = **WH** *be a unique NMF. Given some ϵ* > 0, *there exists a δ* > 0 *such that any nonnegative* **V** = **R** + **N**, where ||**N**||_*F*_ < * δ fulfils*

(18) $$J_{\left(W,H\right)}(W^{\prime },H^{\prime })<\epsilon ,$$

where

(19) $$[W^{\prime },H^{\prime }]=\underset{W^{\prime }{\in R}_{+}^{n\times r},H^{\prime }{\in R}_{+}^{r\times m}}{\mathrm{arg min}}{∥V-W^{\prime }H^{\prime }∥}_{F}.$$

The proof is given in the appendix. The theorem states that if the observation is corrupted by additive noise, then it will result in noisy estimation of **W** and **H**. Moreover, Theorem 6 shows that if the noise is small, it will result in small estimation errors. In this section, the Frobenius norm is used in (17) and (19) to make Theorem 6 concrete. Theorem 6 is also valid with the same proof if any continuous metric is used instead of the Frobenius norm in those equations.

**Example 4.**

**Figure 3:**
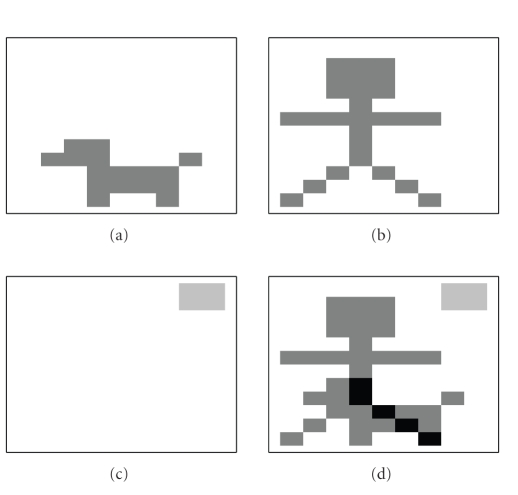
The three basis pictures: (a) a dog, (b) a man, and (c) the sun from Example 4, individually and summed in (d).

This example investigates the connection between the additive noise in **V** and the estimation error on **W** and **H**. The column vectors in **W** are basis pictures of a man, a dog, and the sun as shown in Figures 3(a), 3(b), and 3(c). In Figure 3(d), the sum of the three basis pictures is shown. The matrix **H** is the set of all combinations of the pictures, that is,

(20) $$H=\left[\begin{array}{ccccccc} 1 & 0 & 0 & 0 & 1 & 1 & 1\\ 0 & 1 & 0 & 1 & 0 & 1 & 1\\ 0 & 0 & 1 & 1 & 1 & 0 & 1 \end{array}\right].$$

Theorem 5 can be used to conclude that the NMF of **R** = **WH** is unique because both **W** ^*T*^ and **H** are sufficiently spread and thereby also strongly boundary close.

In the example, two different noise matrices, **N**_*N*_ and **N**_*M*_, are used. The **N**_*N*_ matrix models noisy observation and has elements that are random uniform i.i.d. The **N**_*M*_ matrix contains elements that are minus one in the positions where **R** has elements that are two and zero elsewhere, that is, **N**_*M*_ is minus one in the positions where the dog and the man are overlapping. In this case, the error matrix **N**_*M*_ simulates a model mismatch that occurs in the following two types of real-world data. If the data set is composed of pictures, the basis pictures will be overlapping and a pixel in **V** will consist of one basis picture and not a mixture of the overlapping pictures. If the data is a set of amplitude spectra, the true model is an addition of complex values and not an addition of the amplitudes.

**Figure 4:**
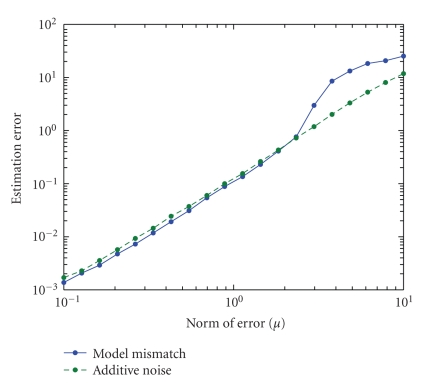
The graph shows the connection between the norm of the additive error ||**N**||_*F*_ and the estimation error of the underlying model *J*_(**W**,**H**)_(**W**′,**H**′). The two noise matrices from Example 4, **N**_*N*_ and **N**_*M*_, are plotted. In this example, the curves are aligned for small errors, and for larger errors, the model error **N**_*M*_ results in much larger estimation errors.

The estimation error of the factorization ***J***_(**W**,**H**)_(**W**′,**H**′) is plotted in Figure 4 when the norm of the error matrix is *μ*, that is, **V** = **WH** + (**N/||N||_F_)** *μ*. An estimate of the [**W**′,**H**′] pair is calculated by using the iterative algorithm for Frobenius norm minimized by Lee and Seung [2]. The algorithm is run for 500 iterations and is started from 100 different positions. The decomposition that minimizes ||**V**−**W**′**H**′||_*F*_ is chosen, and ***J***_(**W**,**H**)_(**W**′,**H**′) is calculated numerically. Figure 4 shows that when the added error is small, it is possible to estimate the underlying parameters. When the norm of added noise matrix increases, the behavior of the two noise matrices, **N**_*N*_ and **N**_*M*_, differ. For **N**_*N*_, the error of the estimate increases slowly with the norm of the added matrix while the estimation error for **N**_*M*_ increases dramatically when the norm is larger than 2.5. In the simulation, we have made the following observation that can explain the difference in the performance of the two types of noise. When **N**_*N*_ is used, the basis pictures remain noisy versions of the man, the dog, and the sun. When **N**_*M*_ is used and the norm is larger than 2.5, the basis pictures are the man excluding the overlap, the dog excluding the overlap, and the overlap of man and dog. Another way to describe the difference is that the rank of **N**_*M*_ is one and the disturbance is in one dimension, where **N**_*N*_ is full rank and the disturbance is in many dimensions. This completes the example.

**Corollary 1.**

*Let* **R** = **WH** *be a unique NMF and* $V=\overset{˜}{W}\overset{˜}{H}$, *where* $\overset{˜}{W}=W+N_{W}$ *and* $\overset{˜}{H}=H+N_{H}$. * Given* **R** *and ϵ* > 0 * there exists a δ* > 0 * such that if the largest absolute value of both* **N**_*W*_ *and* **N**_*H*_ *is smaller than δ*, * then*

(21) $$J_{\left(\overset{˜}{W},\overset{˜}{H}\right)}(W^{\prime },H^{\prime })<\epsilon ,$$

*where* **W**′, **H**′ *are any NMF of* **V**.

**Proof.**

This follows directly from Theorem 6.

The corollary can be used in situations where there are small elements in **W** and **H** but no (or not enough) zero elements—as in the following example.

**Example 5.**

**Figure 5:**
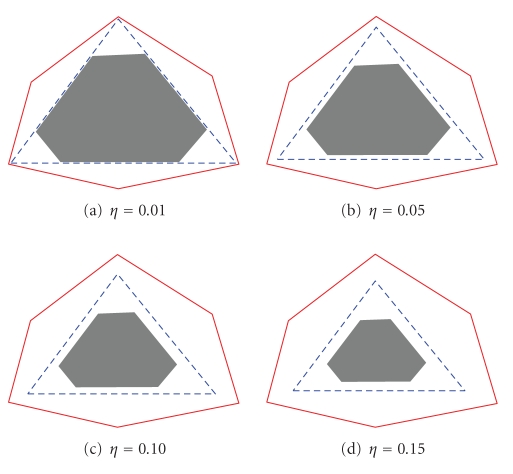
Data constructed as in Example 5 and plotted in the same manner as in Figure 1, that is, the dashed triangle is the desired solution, the solid line is the border of the dual of **H**, and the shaded area is the positive span of **W**. In all the plots, *α* equals 0.3 and *η* equals 0.01, 0.05, 0.1, and 0.15.

Let **R** = **WH**, where **W**, **H** is generated as in Example 3. Let all elements in both **N**_*W*_ and **N**_*H*_ be equal to *η*. In Figure 5, **V** is plotted when *α* = 0.3 and *η* = {0.01, 0.05, 0.10, 0.15}. In this example, neither the shaded area nor the solid border intersect with the desired solution. Therefore, it is possible to get other solutions by simply increasing/decreasing the desired solution. For *η* = {0.01, 0.05}, the corners of the solutions are close to the corners of the desired solution. When *η* = 0.1, the corners can be placed mostly on the solid border and still form a triangle that contains the shaded area. When *η* = 0.15, the corners can be anywhere on the solid border. This completes the example.

## 7. Probability and Uniqueness

In this section, the row vectors of **W** and the column of **H** are seen as results of two random variables. Characteristics of the sample space (the possible outcome) of a random variable that lead to unique NMF will be investigated.

**Theorem 7.**

*Let the row vectors of* **W** *be generated by the random variable 𝒳*_*W*_ *and let the column vectors of* **H** *be generated by a random variable 𝒳*_*H*_. * If the sample space of 𝒳*_*W*_ *is strongly boundary close and the sample space of 𝒳*_*H*_ *is sufficiently spread, then for all ϵ* > 0 * and k * < 1, * there exist N_ϵ_* *and M_ϵ_ such that *

(22) $$\begin{array}{c} p(\underset{D,P}{\min}({∥DPQ-I∥}_{F})<\epsilon )>k, \end{array}$$

*where **Q** is any matrix such that* **WQ** *and* **Q** ^−1^ **H** *are nonnegative and the data size* **R** ∈ ℝ_+_^*n*×*m*^ *is such that n * > *N*_*ϵ*_ *and m * > *M*_*ϵ*_.

**Proof.**

If the data is scaled, **D**_1_ **RD**_2_, it does not change the nonuniqueness of the solutions when measured by the **Q** matrix. The proof is therefore done on the normalized versions of **W** and **H**. Let *𝒴_W_* and *𝒴_H_* be the normalized version of *𝒳_W_* and *𝒳_H_*. There exist finite sets $\overset{¯}{W}$ and $\overset{¯}{H}$ of vectors in the closure of *𝒴_W_* and *𝒴_H_* that are strongly boundary close and sufficiently spread. By Theorem 5, it is known that $\overset{¯}{V}=\overset{¯}{W}\,\;\overset{¯}{H}$ is unique. By increasing the number of vectors sampled from *𝒴_W_* and *𝒴_H_*, for any *ϵ*′ > 0, there will be two subsets of the vectors, **W**′ and **H**′, that with a probability larger that any *k* < 1 will fulfil

(23) $$\epsilon^{\prime }>\;∥\overset{¯}{W}-{W^{\prime }∥}_{F}+∥\overset{¯}{H}-{H^{\prime }∥}_{F}.$$

It is possible to use Corollary 1 on this subset. The fact that limiting min_**D**,**P**_(||**DPQ** − **I**||_*F*_) is equivalent to limiting (21) when the vectors are normalized concludes the proof.

**Example 6.**

**Figure 6:**
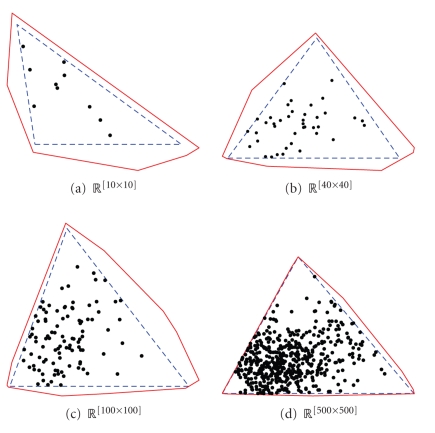
The figure shows data constructed as in Example 6 plotted in the same manner as the previous figure with the exception that each row vector of **W** is plotted instead of the positive span of the vectors. The size of **R** is shown under each plot.

Let all the elements in **H** be exponential i.i.d. and therefore generated with a sufficiently spread sample space. Additionally, let each row in **W** be exponential i.i.d. plus a random vector with the sample space $\left\{\left(\begin{array}{c} 0\\ 1\\ 1 \end{array}\right),\left(\begin{array}{c} 1\\ 0\\ 1 \end{array}\right),\left(\begin{array}{c} 1\\ 1\\ 0 \end{array}\right)\right\}$ and thereby strongly boundary close. In Figure 6, the above variables are shown for the following four matrix sizes **R** ∈ {ℝ^10×10^, ℝ^40×40^, ℝ^100×100^, ℝ^500×500^}. This completes the example.

## 8. Discussion

The approach in this paper is to investigate when nonnegativity leads to uniqueness in connection with NMF, **V** ≈ **R** = **WH**. Nonnegativity is the only assumption for the theorems, and the theorems therefore cannot be used as argument for an NMF to be nonunique if there is additional information about **W** or **H**. An example with stronger uniqueness results is the sparse NMF algorithm of Hoyer [12] built on the assumption that the row vectors in **H** have known ratios between the *L*_1_ norm and the *L*_2_ norm. Theis et al. [13] have investigated uniqueness in this situation and shown strong uniqueness results. Another example is data matrices with an added constant on each row. For this situation, the affine NMF algorithm [14] can make NMF unique even though the setup violates Theorem 3 in this paper.

As shown in Figure 4, the type of noise greatly influences on the error curves. In applications where noise is introduced because the additive model does not hold as, for example, when **V** is pictures or spectra, it is possible to influence the noise by making a nonlinear function on the elements of **V**. Such a nonlinear function is introduced in [15] and experiments show that it improves the results. A theoretical framework for finding good nonlinear functions will be interesting to investigate.

The sufficiently spread condition defined in Section 5 has an important role for unique NMF due to Lemma 3. The sufficiently spread assumption is seen indirectly in related areas where it also leads to unique solutions, for example, in [7] where the groundedness assumption leads to variables with a sufficiently spread sample space. If the matrix **H** is sufficiently spread, then the columns in **W** will occur (almost) alone as columns in **V**. Deville [16] uses the “occur alone” assumption, and thereby sufficiently spread assumption, to make blind source separation possible.

## 9. Conclusion

We have investigated the uniqueness of NMF from three different viewpoints as follows:

- uniqueness in noise free situations;
- the estimation error of the underlying model when a matrix with unique NMF is added with noise; and
- the random processes that lead to matrices where the underlying model can be estimated with small errors.

By doing this, we have shown that it is possible to make many novel and useful characterizations that can be used as theoretical underpinning for using the numerous NMF algorithms. Several open issues can be found in all the three viewpoints that, if addressed, will give a better understanding of nonnegative matrix factorization.

## Appendix

Proof of Theorem 4. The theorem state that **W** = **WI** is a unique NMF. To proof this, it is shown that the condition for Theorem 1 is fulfilled. The positive orthant is self-dual (**I** = **I** ^−1^) and thereby *𝒬* ⊆ *𝒫*, where *𝒬* is an *r*-order simplicial cone that contains span^+^(**W** ^*T*^). Let the set of row vectors in **W** be denoted by *𝒲*. An *r*-order simplicial cone, like *𝒬*, is a closed set and it therefore needs to contain the closure of *𝒲* denoted by $\bar{𝒲}$. The two items in Definition 6 of strongly boundary close can be reformulated for $\bar{𝒲}$ that contains the border:

1. **s** *_n_^j^* = 0 for all *j*,
2. the vectors [**b** ^1^,…, **b** ^*d*−*n*^] are linearly independent.

The rest of the proof follows by induction. If *r* = 2, then $\overset{¯}{𝒲}=𝒫$ and is therefore unique. Let therefore *r* > 2. Then *r* − 1 linearly independent vectors in $\bar{𝒲}$ have zero as the first element, and *r* − 1 of the basis vectors therefore need to have zero in the first element. In other words, there is only one basis vector with a nonzero first element. Let us call this vector **b** ^1^. For all *j* > 1 there is a vector in $\bar{𝒲}$ which is nonnegative in the first element and zero in the *j*th element, so all the elements in **b** ^1^ except the first have to be zero. The proof is completed by seeing that if the first element is removed from the vectors in $\bar{𝒲}$, it is still strongly boundary close and the problem is therefore the *r* − 1 dimensional problem.

Proof of Theorem 6. Let *𝒢* be the open set of all **W**′, **H**′ pairs that are close to **W** and **H**,

(A.1) $$𝒢=\left\{[W^{\prime },H^{\prime }]|\;J_{\left(W,H\right)}(W^{\prime },H^{\prime })<\epsilon \right\}.$$

Let $\bar{𝒢}$ be the set of all nonnegative $\overset{⌣}{W},\;\;\overset{⌣}{H}$ pairs that are not in *𝒢* and where $\max(\overset{⌣}{W},\overset{⌣}{H})\leq \sqrt{1+\max(R)}$. The uniqueness of **R** ensures that

(A.2) $${∥R-\overset{⌣}{W}\overset{⌣}{H}∥}_{F}>0,$$

for all $[\overset{⌣}{W},\overset{⌣}{H}]\in \overset{¯}{𝒢}$. The fact that the Frobenius norm is continuous, $\bar{𝒢}$ is a closed bounded set, and the statement above is positive ensures that

(A.3) $$\underset{[\overset{⌣}{W},\overset{⌣}{H}]\in \overset{¯}{𝒢}}{\min}({∥R-\overset{⌣}{W}\overset{⌣}{H}∥}_{F})=\delta^{\prime }>0,$$

since a continuous function attains its limits on a closed bounded set [17, Theorem 4.28]. The $\overset{⌣}{W},\;\;\overset{⌣}{H}$ pairs that are not in *𝒢* and where $\max(\overset{⌣}{W},\overset{⌣}{H})>\sqrt{1+\max(R)}$ can either be transformed by a diagonal matrix into a matrix pair from $\bar{𝒢}$, $[\overset{⌣}{W}D,D^{-1}\overset{⌣}{H}]\in \overset{¯}{𝒢}$, having the same product $(\overset{⌣}{W}\overset{⌣}{H})$ or it can be transformed into a pair where both $\overset{⌣}{W}$ and $\overset{⌣}{H}$ have large elements, that is,

(A.4) $$\max(\overset{⌣}{W}\overset{⌣}{H})>{\sqrt{1+\max(R)}}^{2}=1+\max(R),$$

and thereby ${∥R-\overset{⌣}{W}\overset{⌣}{H}∥}_{F}>1$. Select *δ* to be *δ* = min(1,*δ*′)/2. The error of the desired solution **R** = **WH** can be bounded by ||**V** − **R**||_*F*_ = ||**N**||_*F*_ < *δ*. Let $\overset{⌣}{V}$ be any matrix constructed by a nonnegative matrix pair not from *𝒢*. Because of the way *δ* is selected, ${∥\overset{⌣}{V}-R∥}_{F}\geq 2\delta$. By the triangle inequality, we get

(A.5) $$\begin{array}{ll} {∥\overset{⌣}{V}-V∥}_{F} & +{∥V-R∥}_{F}\geq {∥\overset{⌣}{V}-R∥}_{F}\\ {∥\overset{⌣}{V}-V∥}_{F} & \geq {∥\overset{⌣}{V}-R∥}_{F}-{∥V-R∥}_{F}\\ & >2\delta -\delta =\delta >{∥V-R∥}_{F}. \end{array}$$

All solutions that are not in *𝒢* therefore have a larger error than **WH** and will not be the minimizer of the error.
